# MicroRNA-Related Polymorphisms in PI3K/Akt/mTOR Pathway Genes Are Predictive of Limited-Disease Small Cell Lung Cancer Treatment Outcomes

**DOI:** 10.1155/2017/6501385

**Published:** 2017-02-09

**Authors:** Wei Jiang, Wenjue Zhang, Lihong Wu, Lipin Liu, Yu Men, Jingbo Wang, Jun Liang, Zhouguang Hui, Zongmei Zhou, Nan Bi, Luhua Wang

**Affiliations:** ^1^Department of Radiation Oncology, National Cancer Center/Cancer Hospital, Chinese Academy of Medical Sciences and Peking Union Medical College, Beijing 100021, China; ^2^Department of Radiation Oncology, Cancer Hospital, Chinese Academy of Medical Sciences, Shenzhen Center, Shenzhen 100021, China

## Abstract

The phosphoinositide-3 kinase (PI3K)/Akt/mammalian target of rapamycin (mTOR) signaling pathway plays an important role in cancer progression and treatment, including that of small cell lung cancer (SCLC), a disease with traditionally poor prognosis. Given the regulatory role of microRNA (miRNA) in gene expression, we examined the association of single nucleotide polymorphisms (SNPs) at miRNA-binding sites of genes in the mTOR pathway with the prognosis of patients with limited-disease SCLC. A retrospective study was conducted of 146 patients with limited-disease SCLC treated with chemoradiotherapy. Nine SNPs of six mTOR pathway genes were genotyped using blood samples. Cox proportional hazard regression modeling and recursive partitioning analysis were performed to identify SNPs significantly associated with overall survival. Three SNPs,* MTOR*: rs2536 (T>C),* PIK3R1*: rs3756668 (A>G), and* PIK3R1*: rs12755 (A>C), were associated with longer overall survival. Recursive partitioning analysis based on unfavorable genotype combinations of the rs2536 and rs3756668 SNPs classified patients into three risk subgroups and was internally validated with 1000 bootstrap samples. These findings suggest that miRNA-related polymorphisms in the PI3K/Akt/mTOR pathway may be valuable biomarkers to complement clinicopathological variables in predicting prognosis of limited-disease SCLC and to facilitate selection of patients likely to benefit from chemoradiotherapy.

## 1. Introduction

Small cell lung cancer (SCLC), which accounts for approximately 13% of lung cancer cases [1], is a common neuroendocrine malignancy characterized by aggressive growth and early metastasis. Chemotherapy with agents of etoposide or topoisomerase 1 inhibitors (irinotecan or topotecan), along with cisplatin and radiotherapy, is the recommended treatment for SCLC with limited disease (LD SCLC) [2, 3]. Unfortunately, there is only a 15% to 20% cure rate and most cases recur rapidly, leading to a very poor overall prognosis [4]. Genetic variations may play an important role in treatment sensitivity; therefore, identification of novel genetic predictors could be helpful for individualized treatment options.

The phosphoinositide-3 kinase (PI3K)/Akt/mechanistic target of rapamycin (mTOR) signaling pathway has been a hot therapeutic target for SCLC in recent research [5]. This pathway is activated in various cancer types, including SCLC [6], and is associated with radiation and chemotherapy resistance [7]. Genetic variations in this pathway are reported to modulate clinical outcomes in patients with esophageal cancer and non-small-cell lung cancer who have undergone chemoradiotherapy [8, 9]. MicroRNAs (miRNAs), a class of small, noncoding RNAs, are key regulators of gene expression in many biological processes [10], including the mTOR pathway [11]. Single nucleotide polymorphisms (SNPs) in miRNA-binding sites may affect the regulatory effect of miRNAs on oncogenes and tumor suppressor genes. However, little published research has considered the impact of miRNA-related genetic polymorphisms in the PI3K/Akt/mTOR pathway and their relationship to SCLC outcome. We therefore analyzed the effect of these genetic variations on the prognosis of patients with LD SCLC receiving curative chemoradiotherapy and selected valuable biomarkers for decision making.

## 2. Materials and Methods

### 2.1. Ethics Statement

This investigation was conducted in accordance with the ethical standards outlined in the Declaration of Helsinki and national and international guidelines. Our institutional review board (Cancer Hospital, Chinese Academy of Medical Sciences) approved this retrospective study, and informed consent was waived.

### 2.2. Study Population

Patients in this study were retrospectively recruited from the Cancer Hospital, Chinese Academy of Medical Sciences, between January 2007 and June 2014. All SCLC were histologically confirmed, staged as LD SCLC based on the International Association for the Study of Lung Cancer (IASLC) classification [12], and initially treated with curative-intent platin-based chemotherapy combined with intensity-modulated radiation therapy (IMRT). Patients who had any other malignancy within five years of enrollment were excluded to avoid a potential confounding effect. Prophylactic cranial irradiation (PCI) was offered to patients who had achieved a complete remission (CR) or partial response (PR), depending on the treating physician's discretion and patient's preference. Clinical information was collected from medical records. Long-term archived serum samples from recruited patients were analyzed [13].

### 2.3. SNP Selection and Genotyping

Candidate miRNA-related SNPs of PI3K/Akt/mTOR pathway genes were selected in two ways: SNPs located at the 3′-UTR of miRNA target genes with a minor allele frequency greater than 0.1 in the Chinese Han population in the PolymiRTS Database 3.0 (http://compbio.uthsc.edu/miRSNP/) and Ensemble Asia database (release 79, http://asia.ensembl.org/index.html?redirect=no) as well as those previously reported in the literature to be associated with cancer were included. Tagging SNPs in linkage disequilibrium were identified using HaploReg version 3 (http://www.broadinstitute.org/mammals/haploreg/haploreg_v3.php) with a cut-off value of* r*^2^ > 0.8.

Genomic DNA was extracted using a TIANamp Blood DNA Kit (TIANGEN Biotech, Beijing, China). Genotyping was conducted using the MALDI-TOF mass spectrometry-based iPLEX Gold assay on the Sequenom MassARRAY Platform (San Diego, CA, USA) and was analyzed using MassARRAY TyperAnalyzer v4.0 software (Sequenom). SNPs with a more than 95% success rate and samples with a call rate exceeding 96% were included. Ultimately, a total of nine tagSNPs in six genes were selected.

### 2.4. Statistical Analysis

Response rates were evaluated according to RECIST 1.0 criteria. The objective response rate (ORR) included CR and PR to treatment. The association of genetic features with ORR was estimated using the Pearson *χ*^2^ test with odds ratios (ORs) and 95% confidence interval (CI). Overall survival (OS) was calculated from pathologic diagnosis to the date of death or last follow-up. The Kaplan-Meier method and log-rank test were used to assess survival for each genotype and clinical characteristics. Hazard ratios (HRs) and 95% CI were estimated by Cox proportional hazards regression models. The adjustment factors included age, gender, smoking history, Karnofsky performance score (KPS), and Charlson comorbidity index (CCI). Genotype analyses were conducted in three genetic models (dominant, recessive, and additive) for each SNP and the model with the smallest *P* value was used. Models with rare genotypes (<5% of patients) were excluded. Recursive partitioning analysis (RPA) was conducted to evaluate the cumulative effects of the genetic variants in the pathway. Concordance probability estimate (CPE) was used to assess the predictive ability of RPA classification [14] from 1000 bootstrap samples.

A two-sided *P* value < 0.05 was considered significant for all statistical analyses. Multiple comparison was performed by Benjamini-Hochberg False Discovery Rate (FDR) correction based on tests for 3 models with a *q*-value of 0.05 [15]. Statistical power of the RPA classification was calculated with an *α* of 0.1. All statistical analyses were carried out using IBM SPSS Statistics 21.0 software (IBM Corp., Armonk, NY) and R version 3.2.3 (http://www.r-project.org.com/).

## 3. Results

### 3.1. Clinical Characteristics

The clinical characteristics of 146 patients with LD SCLC recruited to this study are shown in Table 1. Patients' median age at diagnosis was 56.8 years (range 29–80 years). Of these patients, 91.8% were diagnosed as stage III. Chemotherapy was delivered based on an EP (etoposide + cisplatin) or EC (etoposide + carboplatin) regimen, usually for 2–4 cycles; concurrent treatment was given to 54.8% patients with no difference in overall survival compared to those receiving sequential treatment. A total of 37 patients achieved CR and 84 had a PR. PCI was administered to 43.2% patients, 90.5% of whom had a CR or PR in the primary lesion. The overall median survival time (MST) and 5-year OS rate were 35.1 months and 38.9%, respectively, at a median follow-up time of 42.2 months. Age, KPS, CCI, and PCI were prognostic factors for OS (*P* < 0.05, log-rank test).

### 3.2. Associations of Individual SNPs with Outcome

**Figure 1:**
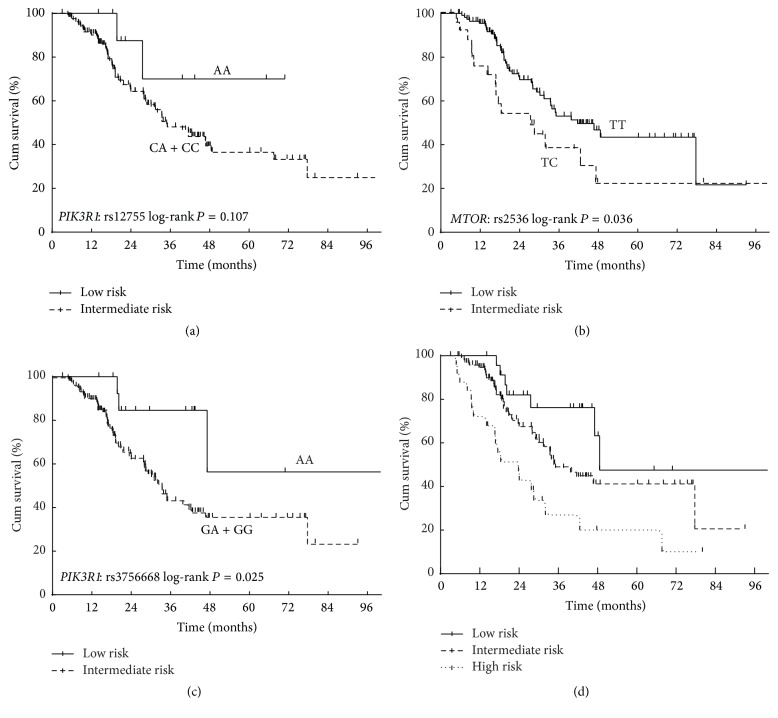
The Kaplan-Meier survival curve of selected SNPs and the RPA classification in patients with limited-disease small cell lung cancer treated with curative chemoradiotherapy. (a)* PIK3R1*: rs12755 (A>C); (b)* MTOR*: rs2536 (T>C); (c)* PIK3R1*: rs3756668 (A>G); (d) RPA classification.

The survival analysis by genotype for each SNP is shown in Table 2. Two SNPs were significantly associated with OS (*MTOR*: rs2536 and* PIK3R1*: rs3756668), rising to three (*PIK3R1*: rs12755) after adjustment for age, gender, KPS, smoking history, and CCI. Patients carrying heterozygous TC of* MTOR*: rs2536 (T>C) had a significantly increased risk of reduced OS compared to those with TT genotype (adjusted HR = 1.948, 95% CI: 1.090–3.482). In addition, variant homozygous genotypes of rs12755 (A>C) and rs3756668 (A>G) in PI3K regulatory subunit 1 (alpha) (*PIK3R1*) were associated with 77.5% (adjusted HR = 0.225, 95% CI: 0.054–0.931) and 61.2% (adjusted HR = 0.388, 95% CI: 0.176–0.856) decrease in the risk of death, respectively (Figures 1(a)–1(c)). However, after multiple comparison correction, the associations with OS of these SNPs were not statistically significant.

Of the three prognostic SNPs, rs12755 and rs3756668 were associated with ORR of chemoradiotherapy in patients with LD SCLC. Individuals carrying the rs12755 C allele showed a significantly higher ORR than those with AA genotype (ORs = 0.908, 95% CI: 0.858–0.961, *P* = 0.037). For rs3756668, carriers of the G allele had a higher ORR compared with the AA genotype (OR = 0.790, 95% CI = 0.720–0.867, *P* = 0.012).

### 3.3. Associations of Individual SNPs with OS in Stage III Patients

Assessing only the 134 patients with stage III SCLC, the overall MST and 5-year OS rate were 35.1 months and 38.2%, respectively.* MTOR*: rs2536 (T>C) and* PIK3R1*: rs3756668 (A>G) remained significantly associated with survival in stage III patients (Table 3).* PIK3R1*: rs12755 (A>C) was a borderline prognostic factor after adjusting for clinical covariates (HR adjusted = 0.242, 95% CI: 0.058–1.010). However, after correcting for multiple comparison, the discriminations of these genetic variants for survival were not statistically significant (Table 3). Other polymorphisms in the PI3K/Akt/mTOR pathway continued not to show significant association with OS.

### 3.4. Recursive Partitioning Analysis (RPA)

To explore the combined effect of unfavorable genotypes, three SNPs significantly associated with OS (rs2536 (T>C) in an additive model, as well as rs12755 and rs3756668 in recessive models), were included in a recursive partitioning analysis. The RPA model was developed based on* PIK3R1*: rs12755 (A>C) and* PIK3R1*: rs3756668 (A>G), and the data set was split into three risk classifications (Table 4). The MST and 5-year OS rate were 48.6, 35.1, and 18.4 months and 47.7%, 40.8%, and 17.6% in the low, intermediate, and high-risk classes, respectively, after adjustment for age, gender, KPS, smoking history, and CCI (Figure 1(d)). The ORs of ORR in the intermediate and high-risk classes were 0.757 (95% CI: 0.679–0.845) and 0.359 (95% CI: 0.236–0.546) using the low risk group as a reference. The statistical power of this classification was 0.74.

### 3.5. Validation of the RPA Classification Using Bootstrap Analysis

Bootstrap analysis was conducted and confirmed a good performance of the RPA predictive model based on PI3K/Akt/mTOR pathway SNPs in survival discrimination by 1000 resampling internal validation datasets (Table 4). The CPE of the RPA classification was 0.69 from 1000 bootstrap samples, supporting the satisfactory predictive efficacy.

## 4. Discussion

SCLC is second only to melanoma in terms of malignancies with a high degree of genomic alternations [16, 17]. As one of the most promising therapeutic targets in SCLC, the PI3K/Akt/mTOR pathway was shown to have a high prevalence of genetic alternations in a comprehensive genomic analysis of SCLC in Asian populations [18]. The PI3K/Akt/mTOR pathway plays a critical role in cancer progression by regulating cell growth, proliferation, and survival [19]. This pathway was also reported to be involved in the development of resistance to radiation and chemotherapy [20–22]. Genetic variants in this pathway were demonstrated to be associated with platinum-based chemotherapy response and prognosis in patients with advanced NSCLC [23]. In this study, we performed a pathway-specific analysis to determine whether polymorphisms in the PI3K/Akt/mTOR pathway may predict outcomes of patients with LD SCLC treated with curative chemoradiotherapy. Three SNPs in miRNA-binding genes in this pathway were identified to be associated with overall survival of these patients, and the unfavorable genotypes of* MTOR*: rs2536 (T>C) and* PIK3R1*: rs3756668 (A>G) were combined by RPA to optimize the prognostic value. To our knowledge, this is the first study to evaluate the pathway-based effect of polymorphisms in the PI3K/Akt/mTOR pathway on clinical outcome of SCLC. The combination of multigenic variants classified these patients into three risk groups and provided a basis to identify patients with LD SCLC that will benefit from chemoradiotherapy.

The* MTOR* gene, located in exon 59 of chromosome 1p36.2, encodes a serine/threonine kinase and emerges as a key downstream effector of the PI3K/Akt/mTOR signaling pathway. Moreover,* MTOR* has been identified as a potential target in SCLC [22]. rs2536 is the mostly frequently studied polymorphism to be associated with cancer susceptibility in the 3′UTR of this gene. However, reports of the association vary in the literature.* MTOR* rs2536 TC/CC genotypes were reported to have an association with decreased risk in acute lymphoblastic leukemia [24], but increased risk in prostate cancer [25], and no association in gastric and esophageal cancer [26, 27]. Therefore, it is possible that the effect of this polymorphism on* MTOR* expression may be cancer type-specific. Until now, there has been no published work on the association between* MTOR*: rs2536 and cancer outcomes. In the present study, patients with rs2536 TC genotype showed a poorer survival than those with a TT genotype. As proposed previously, the binding miRNA-576 at the T allele was substituted for miRNA-767 at the C allele predicted by the bioinformatics web server (https://snpinfo.niehs.nih.gov/snpinfo/snpfunc.php) [25]. Therefore, rs2536 TC genotype was speculated to affect* MTOR* expression, probably because of its effect on affinity for miRNA; however,* MTOR* expression is very complicated. It was found that phosphorylated* MTOR* (*p-MTOR*) was more highly expressed in limited-stage than extended-stage SCLCs, and higher* p-MTOR* expression was associated with better prognosis [28], in contrast to other cancers [29, 30]. Therefore, elucidation of the exact mechanisms requires additional functional studies.

Two SNPs of* PIK3R1*, rs12755 (A>C) and rs3756668 (A>G), were also found to be associated with survival in SCLC. Class IA* PI3K*, composed of a p110 catalytic unit and a regulatory unit, is activated by growth factors and subsequently induces a kinase cascade downstream [31].* PIK3R1* encodes the 85-kD regulatory subunit, which is indispensable for stabilization of the catalytic subunit and downregulating PI3K signaling [32]. A handful of studies showed that polymorphisms of* PIK3R1* were associated with survival in bladder cancer and endometrial cancer [33, 34]. Neither* PIK3R1* SNP discussed in this study has been reported to date. These* PIK3R1* SNPs were found to be associated with favorable survival under a recessive model. Moreover, Ueki et al. [35] demonstrated that heterozygous disruption of* PIK3R1* reduced p85*α* by 50% and enhanced PI3K signaling, while complete depletion of p85*α* in homozygous generated knockout cells significantly decreased PI3-kinase activity. This may be indicative of the functional effect of these loci on* PIK3R1* regulation and activity.

mTOR inhibitors displayed limited antitumor activity in SCLC on account of feedback reactivation of this pathway [36]. Dual inhibition of PI3K and mTOR can block the loop and enhance therapeutic effectiveness [18], and inhibition has been shown to improve tumor radiosensitivity by impairing DNA damage repair and normalizing tumor vasculature [37, 38]. Tumor activity depends on complex signaling networks. Thus, recent studies of cancer-related genetic alternations have also taken a pathway-based approach. In the present work, the combination of two SNPs in* PIK3R1* and* MTOR* provided a more precise discrimination than individual SNPs. Consequently, it is biologically plausible for these potentially functional polymorphisms to have a synergistic effect.

To summarize, this study found three miRNA-related SNPs (*MTOR*: rs2536 (T>C),* PIK3R1*: rs3756668 (A>C) and* PIK3R1*: rs12755 (A>G)) in the PI3K/Akt/mTOR pathway and pathway-based RPA that were associated with survival in patients with LD SCLC treated with chemoradiotherapy. The identification of these predictive factors may be help in selecting the optimal therapeutic strategy for individual patients. However, only nine functional SNPs were selected, based on prior knowledge, for the present study, which means that some important variations in this pathway may have been missed. In addition, multiple correction for these SNPs by 1000 bootstrap samples showed significant associations with OS, suggesting that the negative results after multiple correction were due to the limited number of subjects, although this is the largest study on miRNA-SNPs associated with prognosis in SCLC. Moreover, despite internal validation by bootstrap, there is a lack of independent external validation to confirm our findings. Therefore, additional larger, well-designed studies, including molecular mechanism investigation, are imperative to confirm these findings.

## 5. Conclusions

In conclusion, the current study identified 3 miRNA-related SNPs in the PI3K/Akt/mTOR pathway as prognostic biomarkers for patients with limited-disease SCLC treated with chemoradiotherapy. A risk classification incorporating* MTOR*: rs2536 (T>C) and* PIK3R1*: rs3756668 (A>C) was developed to identify patients likely to benefit from treatment. Replication in a large independent cohort and assessment of biological function is imperative to confirm these findings.

## Figures and Tables

**Table 1:** Clinical characteristics of 146 patients with limited disease-small cell lung cancer.

Variables	*N* (%)	5yOS	*P*
Gender			
Male	104 (71.2%)	33.0%	0.065
Female	42 (28.8%)	47.2%	
Age			
≤60	95 (65.1%)	43.0%	0.015
>60	51 (34.9%)	30.8%	
KPS			
≥90	61 (41.8%)	47.5%	0.009
<90	85 (58.2%)	33.1%	
Location			
Left lobe	69 (47.3%)	39.3%	0.987
Right lobe	77 (52.7%)	37.8%	
Smoking			
Yes	96 (65.8%)	35.7%	0.088
No	50 (34.2%)	40.1%	
Charlson comorbidity index			
≤3	130 (89.0%)	41.7%	0.030
4-5	13 (8.9%)	19.5%	
6-7	3 (2.1%)	0%	
Weight loss			
With	27 (18.5%)	51.3%	0.393
Without	119 (81.5%)	35.2%	
AJCC stage			
IA	1 (0.7%)	100.0%	0.573
IB	2 (1.4%)	0%	
IIA	5 (3.4%)	NA	
IIB	4 (2.7%)	NA	
IIIA	68 (46.6%)	41.7%	
IIIB	66 (45.2%)	34.9%	
Treatment modality			
Concurrent	80 (54.8%)	46.8%	0.401
Sequential	66 (45.2%)	31.2%	
Chemotherapy cycles			
<4	9 (6.2%)	NA	0.382
4–6	126 (86.3%)	40.2%	
>6	11 (7.5%)	38.6%	
Radiotherapy dose			
<60	46 (30.7%)	39.3%	0.525
≥60	100 (69.3%)	39.9%	
PCI			
With	63 (43.2%)	62.1%	1.54*E* − 4
Without	83 (56.8%)	23.1%	

OS: overall survival; AJCC: American Joint Committee on Cancer; PCI: prophylactic cranial irradiation.

**Table 2:** Survival analysis of miRSNPs in mTOR pathway in 146 patients with limited-disease small cell lung cancer.

Gene SNP	Model		5yOS	HR (95% CI)	Log-rank *P*	HR (95% CI) (adjusted)^*∗*^	*P* adjusted^*∗*^	*q*
*VEGFA* rs10434	DOM	GG (100)	39.0%	0.768 (0.449–1.314)	0.334	0.864 (0.498–1.497)	0.601	0.651
		GA + AA (46)	39.7%					
*DDIT4 *rs1053639	DOM	AA (87)	34.0%	0.949 (0.577–1.560)	0.835	0.910 (0.553–1.498)	0.712	0.688
		AT + TT (59)	44.8%					
*MAPK1 * rs1063311	DOM	CC (107)	36.6%	0.637 (0.338–1.199)	0.158	0.546 (0.288–1.037)	0.064	0.165
		CT + TT (36)	45.8%					
*PTEN* rs11202607	ADD	CC (122)	37.2%	0.714 (0.325–1.570)	0.400	0.596 (0.267–1.331)	0.207	0.391
		CT (23)	55.1%					
*PIK3R1 *rs12755	REC	CA + CC (134)	28.7%	0.508 (0.284–0.909)	0.107	0.225 (0.054–0.931)	0.040	0.129
		AA (11)	55.7%					
*MTOR *rs2536	ADD	TT (117)	43.4%	1.838 (1.033–3.272)	0.036	1.948 (1.090–3.482)	0.024	0.103
		TC (25)	22.9%					
*VEGFA *rs3025039	DOM	CC (89)	42.3%	1.025 (0.617–1.705)	0.923	1.083 (0.639–1.837)	0.767	0.704
		CT + TT (55)	35.1%					
*PIK3R1 *rs3756668	REC	GA + GG (119)	35.5%	0.417 (0.190–0.916)	0.025	0.388 (0.176–0.856)	0.019	0.103
		AA (25)	47.7%					
*PTEN *rs701848	DOM	TT (52)	30.2%	0.802 (0.472–1.363)	0.413	0.725 (0.420–1.252)	0.249	0.435
		TC + CC (92)	41.9%					

^*∗*^Adjusted for age, gender, Karnofsky performance score (KPS), smoking history, and Charlson comorbidity index (CCI).

miRSNPs: miRNA-related single nucleotide polymorphisms; OS: overall survival; HR: hazard ratio; CI: confidence interval.

Reference sequences: *VEGFA* GenBank NG_008732; DDIT4 GenBank NM_019058; *MAPK1 *GenBank NG_023054; *PTEN *GenBank NG_007466; *PIK3R1 *GenBank NG_012849; *MTOR *GenBank NG_033239.

**Table 3:** Survival analysis of miRSNPs in mTOR pathway in 134 patients with stage III small cell lung cancer.

Gene SNP	Model		5yOS	HR (95% CI)	Log-rank *P*	HR (95% CI) (adjusted)^*∗*^	*P* adjusted^*∗*^	*q*
*VEGFA* rs10434	DOM	GG (90)	37.9%	0.739 (0.424–1.288)	0.284	0.830 (0.468–1.472)	0.523	0.584
		GA + AA (44)	39.6%					
*DDIT4 *rs1053639	DOM	AA (80)	35.1%	1.042 (0.622–1.744)	0.877	0.970 (0.573–1.639)	0.908	0.709
		AT + TT (54)	42.1%					
*MAPK1 * rs1063311	DOM	CC (97)	36.8%	0.694 (0.366–1.315)	0.260	0.542 (0.308–0.953)	0.101	0.219
		CT + TT (34)	41.7%					
*PTEN* rs11202607	ADD	CC (111)	35.9%	0.607 (0.260–1.416)	0.243	0.493 (0.208–1.169)	0.108	0.225
		CT (22)	58.5%					
*PIK3R1 *rs12755	REC	CA + CC (123)	36.2%	0.360 (0.088–1.475)	0.155	0.242 (0.058–1.010)	0.052	0.161
		AA (10)	70.0%					
*MTOR *rs2536	ADD	TT (108)	43.1%	1.978 (1.087–3.600)	0.026	2.039 (1.117–3.720)	0.020	0.103
		TC (22)	21.2%					
*VEGFA *rs3025039	DOM	CC (80)	42.9%	1.127 (0.666–1.908)	0.655	1.185 (0.687–2.044)	0.541	0.592
		CT + TT (52)	33.4%					
*PIK3R1 *rs3756668	REC	GA + GG (109)	34.0%	0.362 (0.155–0.845)	0.014	0.341 (0.146–0.797)	0.013	0.103
		AA (23)	49.5%					
*PTEN *rs701848	DOM	TT (47)	23.5%	0.885 (0.504–1.556)	0.672	0.759 (0.423–1.362)	0.356	0.488
		TC + CC (85)	40.8%					

^*∗*^Adjusted for age, gender, Karnofsky performance score (KPS), smoking history, and Charlson comorbidity index (CCI).

miRSNPs: miRNA-related single nucleotide polymorphisms; OS: overall survival; HR: hazard ratio; CI: confidence interval.

Reference sequences: *VEGFA* GenBank NG_008732; DDIT4 GenBank NM_019058; *MAPK1 *GenBank NG_023054; *PTEN *GenBank NG_007466; *PIK3R1 *GenBank NG_012849; *MTOR *GenBank NG_033239.

**Table 4:** RPA classification based on unfavorable genotype combinations.

	rs3756668	rs2536	*n*	5y-OS	HR (95% CI) adjusted^*∗*^	*P* adjusted^*∗*^	HR (95% CI) bootstrap	*P* bootstrap
Low risk	AA	Any	25	47.7%	Ref	0.004	Ref	
Intermediate risk	GA + GG	TT	96	40.8%	2.163 (0.963–4.866)	0.062	2.210 (2.154–2.268)	2*E* − 16
High risk	GA + GG	TC	21	17.6%	4.535 (1.813–11.345)	0.001	4.539 (4.409–4.673)	2*E* − 16

^*∗*^Adjusted for age, gender, Karnofsky performance score (KPS), smoking history, and Charlson comorbidity index (CCI).

RPA: recursive partitioning analysis; OS: overall survival; HR: hazard ratio; CI: confidence interval.
